# An investigation of plasma cell-free RNA for the detection of colorectal cancer: From transcriptome marker selection to targeted validation

**DOI:** 10.1371/journal.pone.0308711

**Published:** 2024-08-15

**Authors:** Emmalee J. Northrop-Albrecht, Chung Wah Wu, Calise K. Berger, William R. Taylor, Patrick H. Foote, Karen A. Doering, Anna M. Gonser, Aditya Bhagwate, Zhifu Sun, Douglas W. Mahoney, Kelli N. Burger, Lisa A. Boardman, John B. Kisiel

**Affiliations:** 1 Division of Gastroenterology and Hepatology, Mayo Clinic, Rochester, Minnesota, United States of America; 2 Division of Computational Biology, Mayo Clinic, Rochester, Minnesota, United States of America; 3 Division of Clinical Trials and Biostatistics, Mayo Clinic, Rochester, Minnesota, United States of America; CNR, ITALY

## Abstract

Regular screening for colorectal cancer (CRC) is critical for early detection and long-term survival. Despite the current screening options available and advancements in therapies there will be around 53,000 CRC related deaths this year. There is great interest in non-invasive alternatives such as plasma cell-free RNA (cfRNA) for diagnostic, prognostic, and predictive applications. In the current study, our aim was to identify and validate potential cfRNA candidates to improve early CRC diagnosis. In phase 1 (n = 49; 25 controls, 24 cancers), discovery total RNA sequencing was performed. Select exons underwent validation in phase 2 (n = 73; 35 controls, 29 cancers, 9 adenomas) using targeted capture sequencing (n = 10,371 probes). In phase 3 (n = 57; 30 controls, 27 cancers), RT-qPCR was performed on previously identified candidates (n = 99). There were 895 exons that were differentially expressed (325 upregulated, 570 downregulated) among cancers versus controls. In phases 2 and 3, fewer markers were validated than expected in independent sets of patients, most of which were from previously published literature (FGA, FGB, GPR107, CDH3, and RP23AP7). In summary, we optimized laboratory processes and data analysis strategies which can serve as methodological framework for future plasma RNA studies beyond just the scope of CRC detection. Additionally, further exploration is needed in order to determine if the few cfRNA candidates identified in this study have clinical utility for early CRC detection. Over time, advancements in technologies, data analysis, and RNA preservation methods at time of collection may improve the biological and technical reproducibility of cfRNA biomarkers and enhance the feasibility of RNA-based liquid biopsies.

## Introduction

Colorectal cancer (CRC) is the third most common cause of cancer related death in both men and women in the United States. This year, approximately 153,000 people will be diagnosed with CRC, and around 53,000 will die from the disease [1]. Stage of diagnosis is the most important predictor of survival, with a 91% five-year relative survival for localized disease, and 14% for distant disease [1]. Regular screening can increase early CRC detection. Colonoscopy is the most widely used screening method and has been reported to reduce mortality from CRC [2]. However, overall patient adherence to recommended screening guidelines is inadequate due to absence of insurance, time constraints, household financial burden, bowel preparation, lack of awareness, and fear of procedural risks and pain [3]. Stool based non-invasive testing alternatives include guaiac-based fecal occult blood test (gFOBT), fecal immunochemical test (FIT), and the multi-target stool DNA test (mt-sDNA) [4, 5]. The mt-sDNA test has improved sensitivity for CRCs throughout the colon and high risk precancers compared to fecal blood tests, but sensitivity for the detection of advanced precancerous lesions (43%) has room for improvement [6, 7]. Despite the different screening options available, nearly one third of the screening eligible population is not up to date on current recommendations. The development of blood-based tests would likely further adherence to CRC screening guidelines and revolutionize patient care.

There is a heightened interest in circulating nucleic acids in biofluids as a non-invasive source for cancer biomarkers. Circulating cell-free RNA (cfRNA) is released from both cancerous and non-cancerous cells into the blood by apoptosis, necrosis, or active secretion [8]. Circulating RNA has advantages over circulating DNA and protein. RNA has multiple copies that can provide insights into cellular states and regulatory processes, and it can be quantified in a highly specific and sensitive manner. Cell-free RNA in plasma has been studied in a variety of cancers as a biological marker for early disease diagnosis, survival, surveillance, and recurrence [9, 10]. In 2006, Wong and others were the first to establish beta-catenin mRNA in plasma can serve as a potential marker for CRC [11]. Since then, several studies have investigated cfRNA expression for colorectal precancerous lesions and cancers, but they were often limited by a single gene expression technique being used (RT-qPCR, ddPCR, RNA sequencing, and targeted sequencing) and few if any marker validation steps were performed to confirm their markers’ diagnostic power [12].

While cfRNA is a powerful analyte for cancer research, it is highly unstable and degraded due to high RNase activity in blood unless protected inside extracellular vesicles. Adding to the difficulty, Larson and others (2021) recently reported that ribosomal RNA and mitochondrial RNA make up approximately 97% of cfRNA in plasma, while mRNA was around only 2% [13]. Additionally, technical and biological reproducibility is a concern with cfRNA biomarker discovery [14]. Differences in sample handling/processing methods, data analysis, and external factors such as sex, age, and lifestyle can impact cfRNA expression [15].

In the current study, we aimed to perform a complete investigation into the use of cfRNA in plasma as a source for CRC biomarkers. We not only developed protocols for RNA extraction, library preparation, targeted capture, and reverse transcription-quantitative polymerase chain reaction (RT-qPCR), but also identified a few potential CRC biomarker candidates that need to be further explored. These same optimized procedures may also be useful and applied in other fields of research. Additionally, we uncovered challenges associated with cfRNA data processing and analysis and offer alternative suggestions for future plasma RNA applications.

## Materials and methods

### Study synopsis

**Fig 1:**
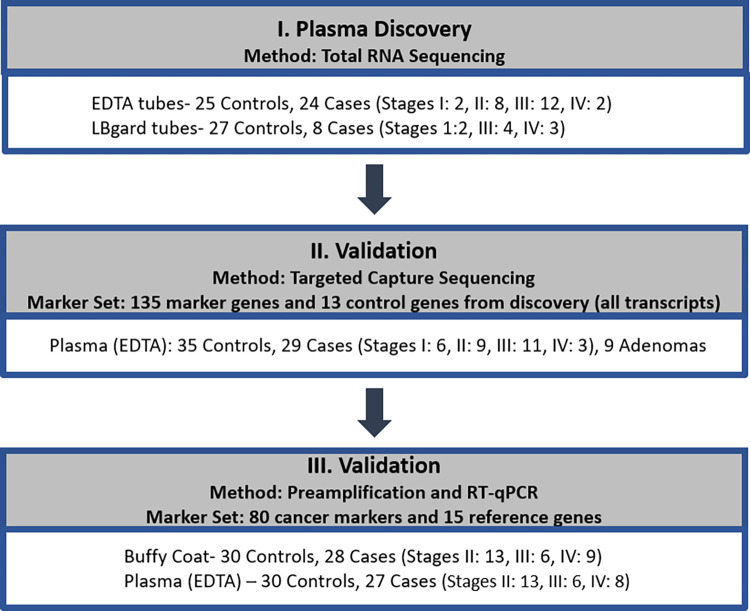
Study flow diagram. Summary of total number of patients within each sample group for the different phases of the experiment.

This work was approved by the Mayo Clinic Institutional Review Board (IRB 18–008752). The patient samples were obtained from another principal investigator, and under the current study there was a waiver of consent. Minnesota authorization was checked prior to obtaining clinical information. The three experiment phases are summarized in **Fig 1**. The first discovery phase was a global approach for identifying differentially expressed RNA markers in plasma from healthy control and cancer patients. During this phase we were also interested in determining the stability of cfRNA from two different collection tube additive types. Next targeted capture sequencing was performed in independent samples to test select markers identified during phase 1. Finally, top performing markers from phases 1 and 2 along with markers identified through literature review were tested on an independent cohort of samples using RT-qPCR.

### Sample selection criteria and processing

Ethylenediaminetetraacetic acid (EDTA) preserved plasma and buffy coat samples were processed and stored according to standardized institutional protocols in the central repository of the Mayo Clinic Biospecimens Accession and Processing laboratory. For a subset of samples in phase 1, whole blood was collected into LBgard® (Biomatrica, San Diego CA) tubes that contained a proprietary stabilizer and processed following the manufacturer’s instructions. Within each phase, control, precancerous lesion, and cancer patients were balanced for age (quartiles), sex, and storage time (median). Cancers included newly diagnosed, referred and regional CRC patients prior to receipt of treatment, enrolled into a research biobank between 2010 and 2022. Controls were recruited from a 7-county regional population and had no history of disease as determined by negative colonoscopy or multi-target stool DNA test. Additionally, for all phases we excluded patients with a history of previous cancer or cancer within 3 years of diagnosis and transplant. Specifically, for phase 2, we also excluded patients with rheumatoid arthritis, lupus, cirrhosis, acute/chronic pancreatitis, pancreatic cysts, or a cancer diagnosis within three years of their blood draw. Patients in the phase 2 study with advanced precursor lesions were equally balanced between adenomatous (tubular adenoma >/ = 1cm or any villous component, or high-grade dysplasia) and sessile serrated lesions >0.5 cm. Statisticians and clinical coordinators had access to detailed clinical information on all the samples during the study (**S1 File).** All samples for each phase were randomized, and the laboratory personal were blinded to sample group information.

### Phase 1-Discovery RNA sequencing and data analysis

Total RNA was extracted from plasma (500ul) using Qiagen’s miRNeasy Serum/Plasma Advanced Kit (Germantown, MD) following the manufacturer’s instructions (n = 84). RNA concentration was quantified using Quant-it RiboGreen (Invitrogen, Waltham MA). Library preparation was performed using the SMARTer Stranded Total RNA-seq Kit-Pico input kit (Takara Bio, San Jose CA), the fragmentation step was skipped. Roche’s KAPA library quantification kit (Indianapolis, IN) and the Agilent Bioanalyzer (Agilent Technologies, Santa Clara, CA) were used to confirm the quality of the libraries. Paired-end sequencing (8 samples per lane) was performed on an Illumina Hiseq 4000 platform at Mayo Clinic’s Genomics Core.

Adapter sequences in raw FASTQ files were trimmed using Trim Galore (https://github.com/FelixKrueger/TrimGalore) and then the trimmed sequences were run through the Mayo Analysis Pipeline MAPS-seq (v3) for RNA sequencing [16]. Reads were aligned to the hg38 genome using Star [17]. The quality of the reads was checked using RSeQC [18]. Differential gene expression analysis using DESeq2 in R (4.1.2) was performed [19]. Exons were considered differentially expressed if the false discovery rate (FDR) ≤ 0.05 and fold change was ≥ 2. Ingenuity pathway analysis was used to explore functions and pathways associated with the differentially expressed exons [20].

### Phase 2- Marker selection, targeted capture sequencing, and data analysis

Total RNA was extracted from EDTA buffered plasma (2 ml) using Qiagen’s QIAamp Circulating Nucleic Acid Kit with DNase treatment and RNA cleanup (Germantown, MD). RNA quantity and quality was assessed using Quant-it RiboGreen (Waltham, MA) and the Agilent Bioanalyzer (Santa Clara, CA). Ribosomal depletion was performed using the NEBNext rRNA Depletion kit v2 Human/Mouse/Rat (New England Biolabs, Ipswich MA). The RNA was then converted into double stranded cDNA using the NEBNext Ultra II RNA First Strand Synthesis with no fragmentation step and NEBNext Ultra II Non-Directional RNA Second Strand Synthesis Modules (Ipswich, MA). Instead of using beads in step two of the second module, the double stranded cDNA was purified using the Zymo Oligo Clean & Concentrator (Zymo Research, Irvine CA). Library preparation was performed using Roche’s KAPA HyperPrep Kit and KAPA Dual-Indexed Adaptor Kit (Indianapolis, IN). Adaptors were diluted to 850nM and incubated for 1 hour. Libraries underwent 19 amplification cycles. For cleanup steps, a higher bead ratio (1.2x) was used to avoid loss of small fragments. Libraries were assessed using Roche’s KAPA Library Quantification kit (Indianapolis, IN) and the Agilent Bioanalyzer (Santa Clara, CA). There were 135 genes selected for further validation in phase 2 based on the following criteria: FDR, fold change, AUC, and GTEx/TCGA data. Many of these genes were previously identified to play a role in cancer development. For normalization purposes, 13 control genes were also selected for probe design. IDT created a custom discovery probe pool with 2x tiling that covered all exons of the genes of interest. The pool originally consisted of 10660 probes, but 289 were removed because the algorithm revealed that they may be present at high levels throughout the genome **(S2 File).** Each library (500ng) was used as input for the hybrid capture reaction using IDT’s Hybridization and Wash Kit (Newark NJ). Each sample underwent 14 cycles of post-capture PCR.

Samples were then run on an Illumina Novaseq SP flow cell with paired-end sequencing at Mayo Clinic’s Genomics Core. The data were processed using MAPS-seq (v3), the same as the discovery dataset [16]. Eight samples with unexpected low number of reads were excluded from the final analysis. Differential expression analyses were conducted using DESeq2 in R (4.1.2) [19]. Exons/genes were considered differentially expressed if the FDR ≤ 0.05 and fold change was ≥ 2.

### Phase 3- Marker selection, plasma RT-qPCR validation, and reference gene testing

For phase 3A, total RNA was extracted from plasma (4 ml) using the QIAamp Circulating Nucleic Acid Kit with DNase treatment and RNA cleanup. Reverse transcription was performed using Applied Biosystems High-Capacity RNA to cDNA kit (Waltham, MA). Samples underwent 14 preamplification cycles using TaqMan Preamp Master Mix (Waltham, MA). Then qPCR was performed in duplicate using TaqMan Fast Advanced Master Mix (Waltham, MA) on a Roche 480 LightCycler. A no template control was present on each plate to assure there was no background contamination. TaqMan assays (n = 99) were selected based on candidates identified from discovery (phase 1), targeted sequencing (phase 2), TCGA, and published literature (**S3 File**). The reference gene with the lowest variability across samples was used as a normalizer for targeted genes of interest (delta CT).

RNA inputs into cDNA synthesis were not the same among plasma samples because of the variation and low yields across patients. Therefore, buffy coats were also used to determine the stability of 15 reference genes. For phase 3B, total RNA was extracted from 250ul of buffy coat using the Qiagen’s miRNeasy Mini Kit with DNase treatment and RNA cleanup (Germantown, MD). Total RNA (430ng) was reverse transcribed using Applied Biosystems High-Capacity RNA to cDNA kit (Waltham, MA). The cDNA was diluted to a total volume of 90ul. Then qPCR was performed in duplicate using TaqMan Fast Advanced Master Mix on a Roche 480 LightCycler (Indianapolis, IN) following the manufacturer’s recommended PCR conditions. A no template control was present on each plate to assure there was no background contamination. Standard deviations of Ct values were calculated among cancer and control patients. The most stable assay across patients was identified and applied to the plasma data for normalization.

### Statistical methods

Sample size for RNA sequencing were based on the work of Hart and others (2013) [21]. The calculation of sample size requires an estimate of biological variation (BV). From their results on tissue experiments, they found that the median BV was 0.63 and the 90^th^ percentile was approximately 0.9. To be conservative, we used the 90^th^ percentile estimate. Assuming an average read depth of 20 reads per subject for each transcript, in order to detect a fold change of 2 or higher with 80% power with a two-sided significance level of 0.05 required a minimum of 19 patients per group. Larger group sizes or lower BV for an individual transcript would lower the detectable fold-change given the same power and two-sided significance level.

For phase 1, unsupervised clustering was performed using all expressed genes with 1-Pearson correlation as distance matrix and ward.D2 agglomeration method in R. For phase 2, Kruskal–Wallis (non-parametric) tests were used to compare two or more groups for the RNA yield data. This data was analyzed and plotted in GraphPad Prism 9.5.1 (Carlsbad CA). For phase 3, differential expression between groups (cancers and controls or different cancer stages) ANOVA test with TukeyHSD for contrasts in R (v4.1.2) was performed. Relative fold change was calculated by the 2^-ΔΔCt^ method.

## Results

### Identification of plasma cfRNA candidates by total RNA sequencing (phase 1)

**Fig 2:**
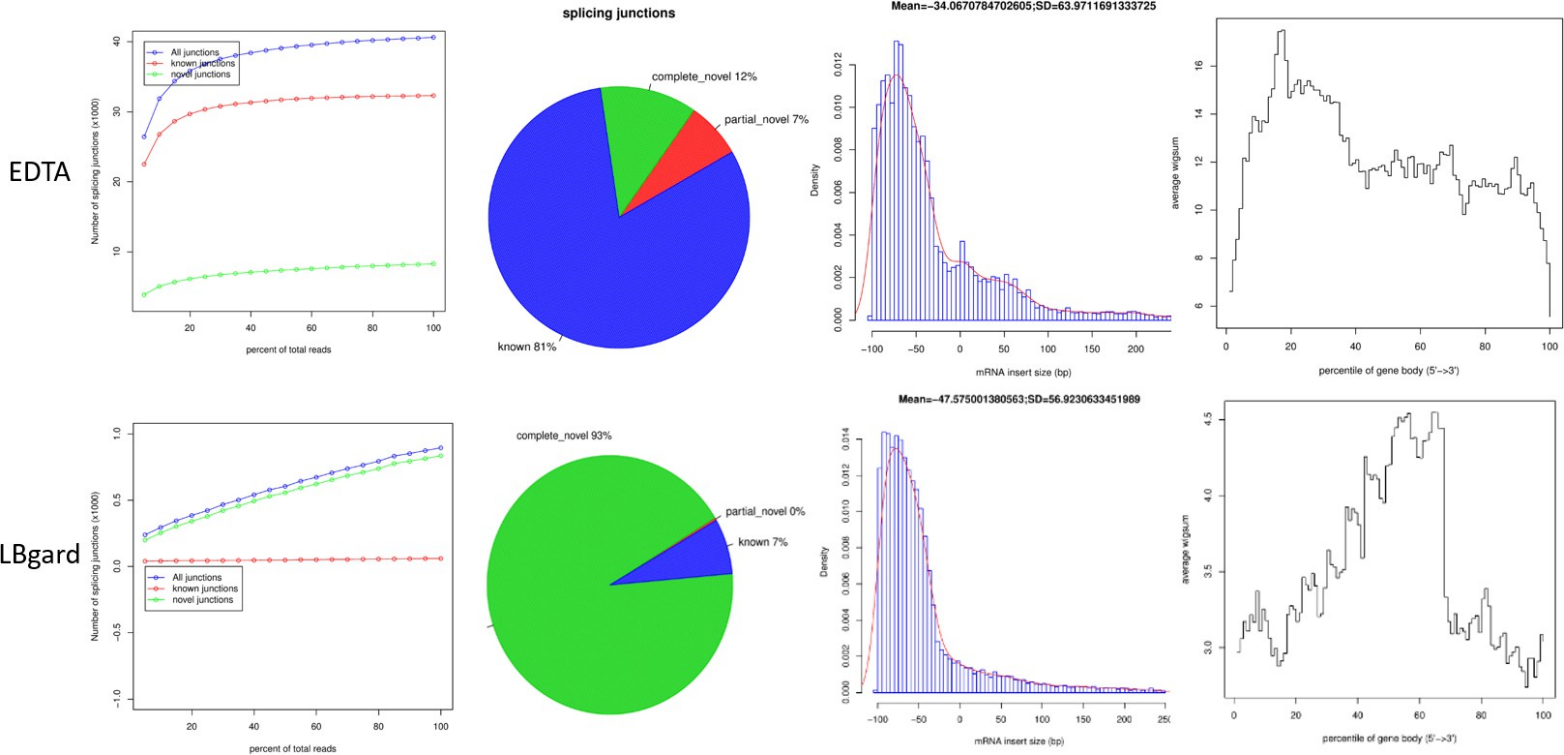
Comparison of RSEQC results from two plasma tube types (phase 1). Samples from LBgard tubes consistently had poor quality metrics (low mapping rates and low number of reads mapped to annotated genes) compared to EDTA preserved samples.

**Fig 3:**
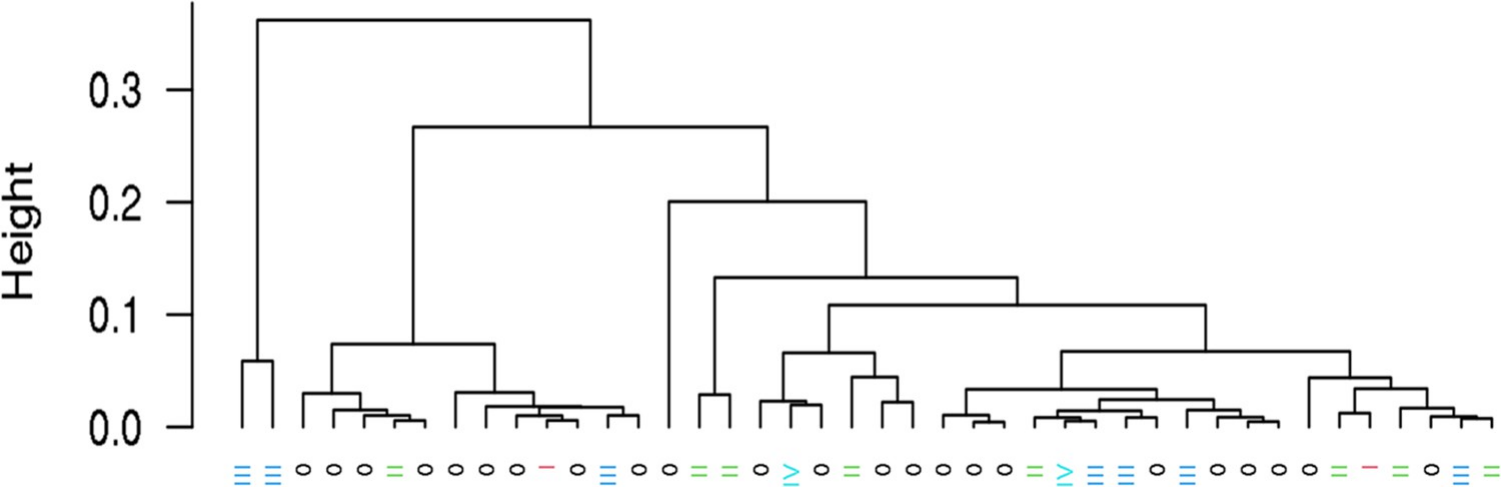
Unsupervised clustering of expressed genes dendrogram (phase 1). There was no clear separation between cancers and controls and between different stages of cancer (0 for controls) although in certain sub-clusters cancers and controls were separated.

There was a total of 84 plasma samples that underwent total RNA sequencing in order to identify potential cfRNA targets that distinguish cancer from control patients. The proprietary reagent in LBgard tubes (n = 35) appeared to be insufficient at stabilizing cfRNA because these samples consistently had poor quality metrics (low mapping rates and low number of reads mapped to annotated genes) and were therefore removed from the final analysis (**Fig 2**). Additionally, there were 7 EDTA buffered plasma samples removed due to poor quality. The poor results observed in some of these samples was likely due to insufficient starting material prior to library preparation. For the samples that passed QC metrics [n = 42: 23 controls, 19 cancers (stages I: 2, II: 8, III: 7, IV: 2)] there was an average of approximately 65.4M reads per sample, with a range of 5.2M to 83.1M (**S4 File**). On average, 81% of reads mapped to human genome with a range of 50% to 89% (**S4 File**). Unsupervised clustering for expressed genes did not see clear separation between cancers and controls and between different stages of cancer (the labels below dendrogram, the 0 for controls) although in certain sub-clusters cancers and controls were separated (**Fig 3**). Since the cell-free RNA data were highly fragmented (uneven gene body coverage, shorter fragments generally less than 100bps, and low transcript integrity number [most TINs <20, which represents percentage of transcripts with uniform read coverage]), we opted to conduct differential exon analysis, which could help localize the changed region in a gene. There were 895 cfRNA exons that were differentially expressed among cancer and control patients (**S4 File**). There were 570 exons (264 genes) downregulated and 325 exons (189 genes) upregulated in the cancer group.

### Pathway analysis for differentially expressed genes (phase 1)

Ingenuity Pathway Analysis (IPA) was used to explore the functions of differentially expressed genes among cancer and control patients (**S5 File**). Within the top ten ingenuity canonical pathways were: protein kinase A signaling, molecular mechanisms of cancer, thrombin signaling, integrin link kinase signaling, alpha adrenergic signaling, and IL-8 signaling, all of which have been reported to impact carcinogenesis. The top three categories for the diseases and disorders analysis were cancer, gastrointestinal disease, and organismal injury and abnormalities. Top molecular and cellular functions were cellular assembly and organization, cellular function and maintenance, cellular movement, molecular transport, and cellular development.

### Plasma RNA composition (phase 2)

**Fig 4:**
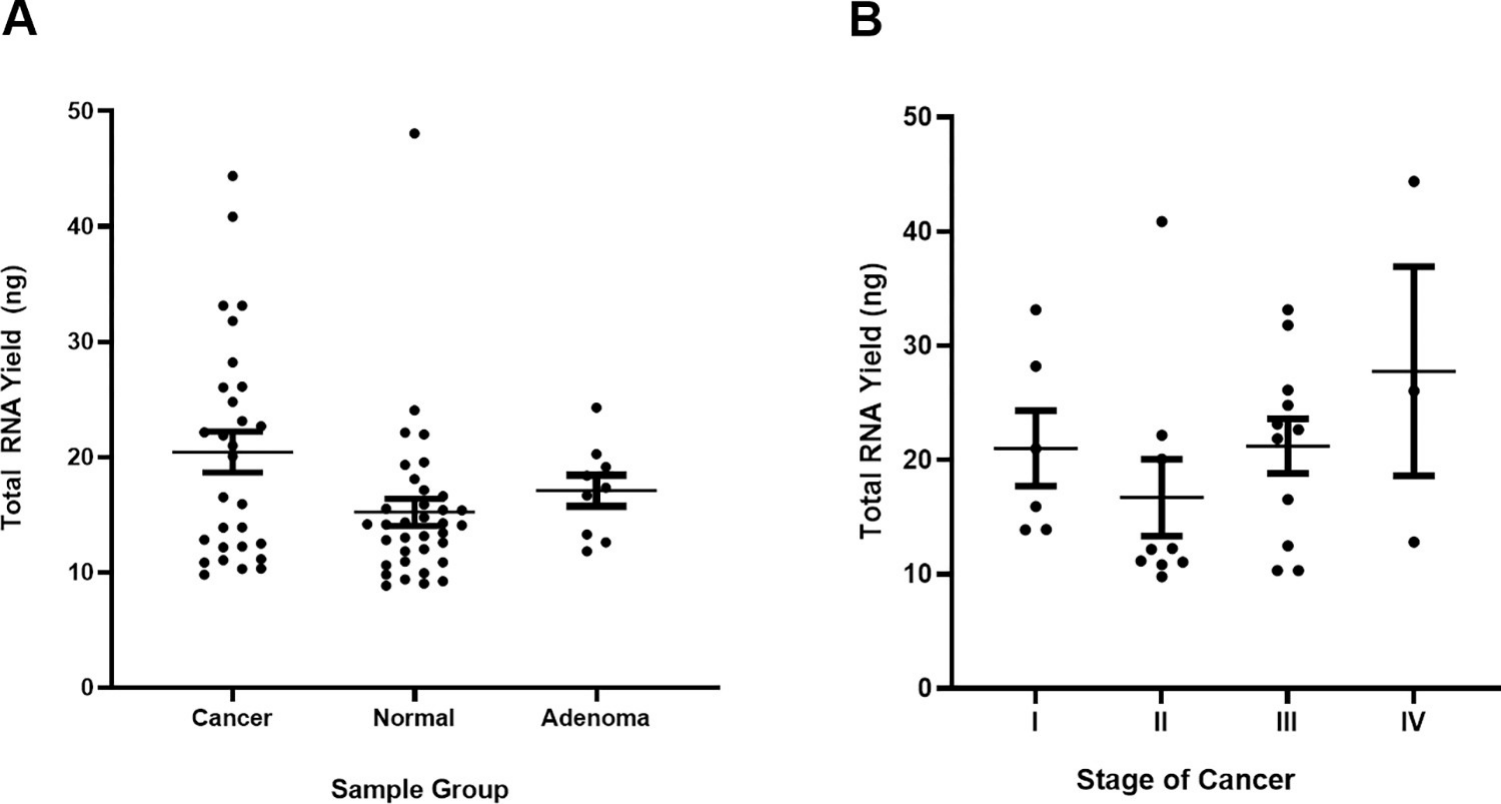
Plasma RNA composition (phase 2). (a) Plasma RNA yield (ng) for the different sample groups. There was a tendency for cancer patients to have increased cfRNA yields, Kruskal-Wallis test (Mean ± SEM) (b) Plasma RNA yield (ng) by cancer stage. There was no difference in total RNA yield among different stages of cancer, Kruskal-Wallis test (Mean ± SEM).

For phase 2, plasma RNA yield was compared among sample groups (n = 73). The median RNA yield was 20.06 ng, 14.15 ng, and 17.32 ng in cancer, control, and adenoma plasma samples, respectively (**Fig 4A**). There was a tendency for cancer patients to have increased cfRNA yields compared to control patients (*P* = 0.06). There was no significant difference in total RNA yield among the different stages of cancer (*P* > 0.44; **Fig 4B**). For most plasma samples, RNA integrity number (RIN) was between 2.0–2.5.

### Validation of cfRNA candidates using targeted capture sequencing (phase 2)

Through extensive troubleshooting we developed a protocol for ribosomal rRNA depletion, double stranded cDNA generation, library preparation, and hybrid capture. There were 73 EDTA plasma samples that were processed for targeted sequencing. Eight samples failed library prep and were therefore removed prior to data analysis of the remaining 30 controls, 9 adenomas, and 25 cancers (stages I: 5, II: 8, III: 10, IV: 2). There was an average of approximately 13.2M reads per sample, with a range of 9.1M to 19.6M (**S6 File**). On average, 96% of reads mapped to human genome with a range of 84% to 98% (**S6 File**). The average number of reads that mapped to targets was 72% with a range of 31% to 83% (**S6 File**).

**Fig 5:**
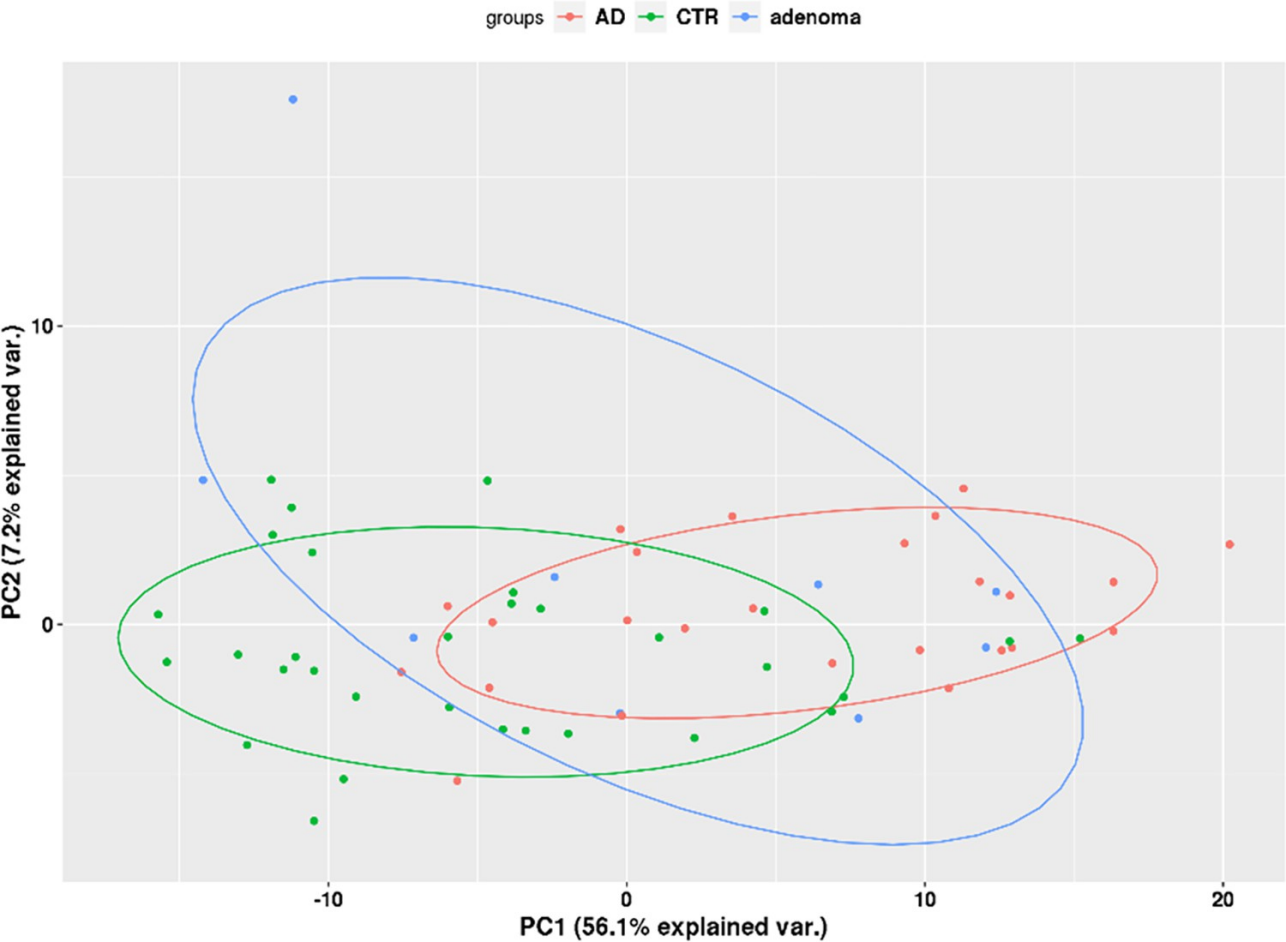
Targeted capture sequencing principal component analysis plot (phase 2). There was a lack of clustering observed among sample groups (AD/Cancer: red, adenoma: blue, and CTR/control: green).

For the adenoma versus control patient comparison, there were 13 differentially expressed exons (7 downregulated, 6 upregulated; **S6 File**). For the cancer versus control comparison, there were 224 differentially expressed exons (85 downregulated, 138 upregulated; **S6 File**). For the cancer versus adenoma comparison, there were 12 differentially expressed exons (1 downregulated, 11 upregulated; **S6 File**). When comparing cancer versus control on a gene level, there were 35 differentially expressed genes, all of which were downregulated in cancer patients (**S6 File**). Overall, there were no differentially expressed exons/genes that were validated from the discovery phase among cancer and control groups. Furthermore, the principal component analysis (PCA) plot for all exons revealed a lack of clustering among the three sample groups (**Fig 5**).

### Validation of cfRNA candidates using RT-qPCR (phase 3)

As a final experiment, we wanted to further determine if we could validate the cfRNA candidates identified from phase 1, phase 2, TCGA, and previously published literature using an alternative quantification method, RT-qPCR **(S3 File).** Patient demographics for this phase are presented in **Table 1**. Given the low RNA yield in plasma and the number of markers, preamplification was necessary. For plasma samples [n = 57; 30 controls, 27 cancers (stages II: 13, III: 6, IV: 8)], RPS12 had the lowest standard deviation in controls (**S1 Fig**). For buffy coat samples [n = 58; 30 controls, 28 cancers (stages II: 13, III: 6, IV: 9)], CASC3 had the lowest standard deviation (**S2 Fig**).

**Table 1:** Clinical characteristics of cancer and control patients for phase 3 study.

Characteristics	Cancer Patients (n = 27)	Controls (n = 30)
**Age [median(range)]**	62 (35–84)	61(28–89)
**Sex (Male/Female)**	16/11	16/14
**Smoking Status** *		
Never	16	14
Current	2	4
Past	9	12
**Alcohol Use**		
Yes	14	22
No	13	8
**Site**		
Left-sided	21	
Right Sided	5	
Unknown	1	
**Size (cm; range: 2.1–11)** **		
≤5.20	8	
>5.2	7	
Unknown	12	
**Stage**		
II	13	
III	6	
IV	8	

* Current: currently or quit in last 3 months, Past: past use, but not in last 3 months

** If multiple tumors present, median size was used

For the first analysis we used RPS12 as the normalizing gene and calculated the delta Ct value for each marker of interest. A simple nonparametric t test was performed on the delta delta Ct values. The expression of most markers was increased in plasma samples from cancer patients compared to control patients, however only six previous targets were statistically significant and validated (*P* ≤ 0.05; **Table 2**, **S7 File**). Of the differentially expressed TaqMan assays in phase 3, five (FGB [n = 2], RPL23AP7 [n = 2], GSDMA) were selected based on literature search and one (GPR107) was previously identified in the current study. Differential expression among the different cancer stages revealed eight differentially expressed TaqMan assays among stage IV patients and control patients (**S7 File**). Of the differentially expressed TaqMan assays, five (FGA, FGB [n = 2], CFAP58 [n = 2]) were selected based on literature search, two (PERP, GPR107) were selected from the current dataset, and one (CDH3) was selected from TCGA analysis. The second analysis used CASC3 as the normalizing gene, and only identified two TaqMan assays (RPL8, TMBS10) that were classified as statistically significant (**S8 File**), and they were both downregulated in cancer patients.

**Table 2:** Differentially expressed transcripts validated by RT-qPCR.

Transcript	Reference Gene	Adjusted P value	Fold Change
FGB_5942	RPS12	0.025	2.21
GPR107	RPS12	0.029	1.30
RPL23AP7_7498	RPS12	0.031	2.65
GSDMA_7851	RPS12	0.033	3.93
FGB_5941	RPS12	0.036	2.24
RPL23AP7_4782	RPS12	0.043	4.50
RPL8_1285	CASC3	0.017	-1.23
TMBS10_0508	CASC3	0.022	-1.26

## Discussion

The unstable nature of RNA makes it a difficult cancer biomarker class to study, especially for cfRNA in plasma since high RNAse activity leads to poor RNA quality and quantity. Circulating mRNAs were first reported in cancer patients in the 1990s, however, there are no cell-free RNA tests that are FDA approved to date [22–24]. Previous research has examined the use of cell-free mRNA markers in colorectal adenomas and/or cancers but prior to the work presented here, a comprehensive investigation performing discovery followed by multiple validation steps has not been conducted [25–27].

Our initial total RNA sequencing experiment revealed hundreds of possible RNA targets, 360 exons of which were upregulated in colon cancer patients compared to controls. We further narrowed down the list of the strongest candidates (n = 135 genes) to pursue validation in an independent cohort. The second experiment used targeted capture sequencing for validation, and of the 35 differentially expressed genes detected, all were downregulated in the colon cancer group compared to controls, in contradistinction to the findings of our initial discovery experiment. Similarly, the control genes we selected based on the initial discovery phase experiment were not stable across conditions. Overall, none of the selected markers identified among cancer and controls in phase 1 were validated in the phase 2 study.

Additionally, patients with precancerous lesions were added to phase 2. Current non-invasive screening options have less sensitivity for detecting adenomas and sessile serrated lesions than for cancers. The early detection and removal of precancerous polyps remains critical to interrupt the adenoma-carcinoma sequence and prevent the development and spread of CRC [28]. In the current dataset, there were only a few differentially expressed targets when comparing adenoma patients to either control or cancer patients, none of which appeared to have strong potential as biomarkers. The limited ability to detect advanced precursor lesions from blood plasma liquid biopsies has also been noted for cell-free DNA as well [29].

The third experiment, we used an alternative approach, RT-qPCR, to attempt to validate markers identified from phase 1 (discovery total RNA sequencing), phase 2 (targeted capture sequencing), literature, and GTEx/TCGA analyses. Overall, we identified six transcripts that were upregulated and validated among cancer and control patients. These markers were selected from previously published data (FGB, RP23AP7, GSDMA; [30]) and phase 2 of the current dataset (GPR107). When performing pairwise comparisons among the different stages of cancer, stage IV patients had increased expression of FGA, FGB, PERP, GPR107, CDH3 compared to controls. This is likely due to an increase in tumor burden and release of more nucleic acids into circulation [31, 32]. Additionally, we determined that results differed based on the reference gene that was used to normalize the data. An alternative approach would be to use digital droplet PCR. This recently developed technology has been shown to have higher precision and reproducibility compared to conventional qPCR [33].

Specifically, CDH3 functions in the regulation of cell adhesion and is likely involved in the progression and metastasis of colorectal cancer. CDH3 mRNA was upregulated in colon adenocarcinoma tissue compared to normal colon tissue [34, 35]. Additionally, based on a TCGA data, a cutoff was established, and high CDH3 expression had a better prognosis for patients compared to low/medium expression [35]. Fibrinogen is a protein that is encoded by three different genes (FGA, FGB, FGG), two of which were upregulated in plasma samples from cancer patients in the current study. It regulates the expression of genes involved in cell cycle regulation and metabolism by activating the focal adhesion kinase pathway, leading to the destabilization of p53 causing tumor growth and limiting senescence [36]. At the protein level, higher levels of fibrinogen in plasma have been strongly associated with poor prognosis and lower survival rate in many cancers including colorectal [37, 38].

There are multiple factors that may have contributed to lack of marker validation in the current study. Samples in the three phases comprised of different patients so the validation was totally independent due to limited plasma volumes and RNA yield/quality. There were also differences in volume of plasma used across experiments (phase 1: 500ul, phase 2: 2ml, phase 3: 4ml) due to changes in RNA extraction kit protocols and plasma availability. Furthermore, since the experiments were carried out across several years there were changes in technologies, RNA extraction, and library preparation kits used. For the initial discovery total RNA sequencing experiment, sample size was smaller than expected due to more poor-quality samples than anticipated and differential expression data was not statistically strong which may have led to inferior marker identification for downstream validation steps. Additionally, deeper sequencing may have revealed additional promising candidates. Finally, there may be a lack of universal RNA markers across patients. Given the highly fragmented nature of RNA in plasma, a possible alternative would be to focus on RNA within extracellular vesicles due to their protection from enzymatic degradation [39]. Another alternative for studying early colorectal cancer precursors, would be to focus on the abundant release of RNA by luminal exfoliation into stool directly, because it occurs much earlier than vascular invasion into the blood [40].

Cell- free RNA biomarkers have low reproducibility caused by technical and biological variability, lack of standardized methods, and insufficient sample sizes [41, 42]. We encountered similar issues in the current investigation and only validated a small number of potential cfRNA markers for CRC detection. It may be beneficial to further explore these cfRNA candidates (FGA, FGB, GPR107, CDH3, RP23AP7). Additionally, our rigorous protocol optimization and data analyses may be applied to other areas of research beyond just the scope of CRC detection. We hypothesize that over time improvement in technologies, data analysis, and RNA preservation methods at the time of blood collection may improve the feasibility of an RNA- based liquid biopsy in the future.

## Supporting information

S1 File Sample/patient information. (XLSX)

S2 File Phase 2: Targeted capture sequencing IDT probes. (XLSX)

S3 File Phase 3: RT-qPCR assays. (XLSX)

S4 File Phase 1: RNA sequencing metrics and differential gene expression analysis. (XLSX)

S5 File Phase 1: Ingenuity pathway analysis. (XLSX)

S6 File Phase 2: Targeted capture sequencing metrics and differential gene expression analysis. (XLSX)

S7 File Phase 3: RT-qPCR data analysis results (RPS12 reference gene). (XLSX)

S8 File Phase 3: RT-qPCR data analysis results (CASC3 reference gene). (XLSX)

S1 Fig Plasma mean Ct values for each reference gene for cancers and controls (phase 3A). Among all reference genes, RPS12 and CASC3 had the lowest standard deviation, while ACTB and APP had the largest standard deviation.
(TIF)

S2 Fig Buffy coat mean Ct values for each reference gene for cancers and controls (phase 3B). Among all reference genes, CASC3 had the lowest standard deviation, while UBB had the largest standard deviation.
(TIF)
