# Supplementary figures and images for: An investigation of plasma cell-free RNA for the detection of colorectal cancer: From transcriptome marker selection to targeted validation
Source: PLoS One. 2024 Aug 15;19(8):e0308711. doi: 10.1371/journal.pone.0308711 (PMC11326608; doi:10.1371/journal.pone.0308711)

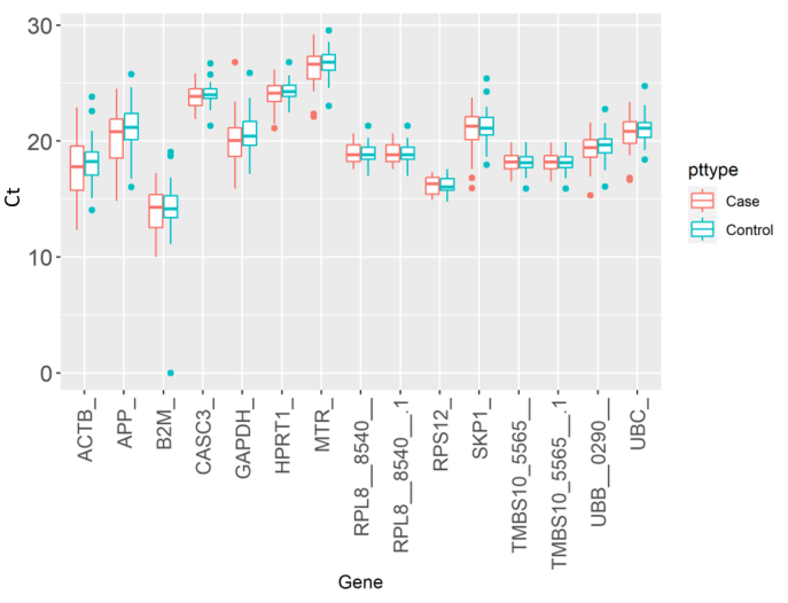

Supplement: S1 Fig — Among all reference genes, RPS12 and CASC3 had the lowest standard deviation, while ACTB and APP had the largest standard deviation. (TIF) [file pone.0308711.s009.tif]

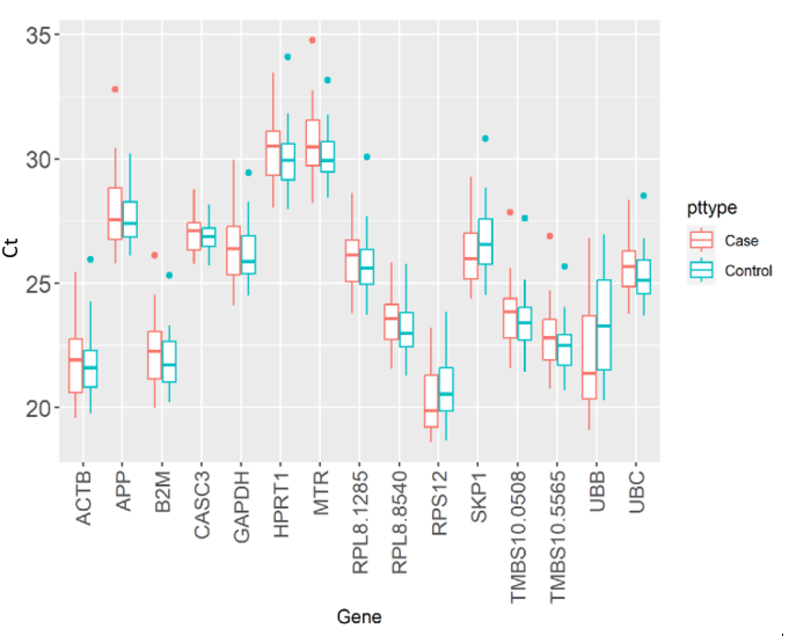

Supplement: S2 Fig — Among all reference genes, CASC3 had the lowest standard deviation, while UBB had the largest standard deviation. (TIF) [file pone.0308711.s010.tif]
